# Early detection and persistent positivity of anti-*Leishmania* antibodies using a recombinant protein-based ELISA in naturally infected dogs in Brazil

**DOI:** 10.1186/s13071-021-04895-z

**Published:** 2021-08-12

**Authors:** Matheus Silva de Jesus, João Victor Andrade Cruz, Lívia Brito Coelho, Lairton Souza Borja, Edmilson Domingos da Silva, Manuela da Silva Solcà, Claudia Ida Brodskyn, Deborah Bittencourt Mothé Fraga

**Affiliations:** 1 Instituto Gonçalo Moniz-Fundação Oswaldo Cruz, Rua Waldemar Falcão 121, Bahia, Salvador Brazil; 2 Instituto de Tecnologia em Imunobiológicos, Bio-Manguinhos, Rio de Janeiro, RJ Brasil; 3 Escola de Medicina Veterinária e Zootecnia-Universidade Federal da Bahia, Av. Adhemar de Barros 500, Bahia, Salvador Brazil; 4 Instituto Nacional de Ciência e Tecnologia-Investigação em Imunologia/INCT-III, São Paulo, São Paulo Brazil; 5 Instituto Nacional de Ciência e Tecnologia de Doenças Tropicais/INCT-DT, Bahia, Salvador Brazil

**Keywords:** Early diagnosis, Canine leishmaniasis, rLci5, Diagnostic persistence

## Abstract

**Background:**

Visceral leishmaniasis (VL) is a zoonotic disease caused by *Leishmania infantum*, for which dogs constitute the main urban parasite reservoir. Control measures and the treatment of canine visceral leishmaniasis (CVL) are essential to reduce VL cases. Early and accurate detection of *L. infantum*-infected dogs is crucial to the success of VL control. To improve the serological detection of *L. infantum*-exposed dogs, we evaluated the early diagnosis capacity of a recombinant protein (rLci5) in an immunosorbent assay (ELISA) to detect naturally infected dogs. Additionally, we evaluated the persistence of the positive results obtained by rLci5 ELISA in comparison to other conventional diagnostic test methods.

**Methods:**

Serum samples obtained from 48 *L. infantum*-infected dogs involved in a cohort study were evaluated using different diagnostic methods (qPCR, EIE-LVC, DPP-LVC and splenic culture). The results were compared to rLci5 ELISA to determine its capacity to diagnose *L. infantum* infection at earlier infection time points. The persistence of positive diagnostic test results was also compared for each dog evaluated.

**Results:**

rLci5 ELISA presented higher rates of positive results at early time points compared to the other diagnostic tests employed in the cohort study, as early as 24 months prior to detection by other tests. rLci5 ELISA positivity was 52.1% (25/48) at baseline, while qPCR was 35.4% (17/48), DPP-LVC 27.1% (13/48), EIE-LVC 22.9% (11/48) and culture only 4.2% (2/48). In at least one of the time points of the 24-month cohort study, rLci5 ELISA was positive in 100% (48/48) of the dogs, versus 83% (40/48) for qPCR, 75% (36/48) for DPP-LVC, 65% (31/48) for EIE-LVC and 31% (15/48) for culture. Investigating clinical signs in association with diagnostic test positivity, rLci5 ELISA successfully detected CVL in 62.9% (95/151) of the clinical evaluations with a score of 0–3, 64.3% (45/70) with scores between 4 and 7, and 73.7% (14/19) with scores > 7, providing higher rates of positivity than all other methods evaluated. Moreover, rLci5 ELISA presented the greatest persistence with respect to test positivity: 45.8% of the dogs evaluated.

**Conclusion:**

Four diagnostic tests were compared to rLci5 ELISA, which presented earlier infection diagnosis and a greater persistence of positive test results. Accordingly, the use of the rLci5 ELISA can improve CVL diagnostic performance by detecting infected dogs sooner than other testing methods, with enhanced persistence of positive results over the course of the infection.

**Graphic abstract:**

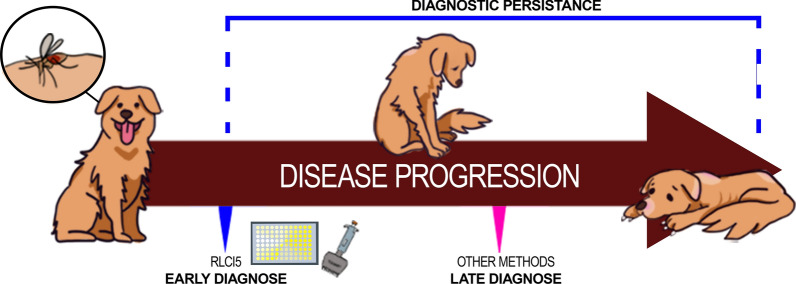

**Supplementary Information:**

The online version contains supplementary material available at 10.1186/s13071-021-04895-z.

## Background

The protozoan parasite *Leishmania infantum* is the main etiological agent of human visceral leishmaniasis (VL) in Brazil, an endemic zoonosis commonly found in tropical countries [1]. According to the Brazilian Ministry of Health, ~ 3500 cases are registered annually throughout the country [2]. In Brazil, the sand fly *Lutzomyia longipalpis* is the main vector of *L. infantum* [3]. Patients with VL may develop clinical signs including prolonged fever, anemia, weight loss, weakness, hepatomegaly and splenomegaly [4].

Dogs are the main urban reservoirs of *L. infantum*, which may play an important role in the transmission and maintenance of VL, resulting in the accidental infection of humans [4, 5]. Early and effective diagnosis is essential to establishing efficient control measures [6, 7]. Among the available diagnostic methods, serological assays offer several advantages including low cost, feasibility in field settings and rapid test results, as well as adequate parameters of sensitivity and specificity when appropriate antigens are employed [8].

Recombinant proteins, which have been used as antigens to improve immunodiagnostic performance, can be identified using proteomics and bioinformatics technology [7]. Machado et al. [9] demonstrated significant diagnostic results using recombinant proteins and synthetic peptides, with very high sensitivity and specificity for diagnosing VL in humans and dogs. In addition, Santos et al. [10] found that *L. infantum* elongation factor-1 beta protein and its recombinant version both offered high sensitivity and specificity for the detection of anti-*Leishmania* antibodies in asymptomatic and symptomatic dogs. These same proteins may also be useful as prognostic markers in human sera, since they are able to detect reductions in antibody levels following treatment in humans [10].

Our group previously identified recombinant antigens for use in canine visceral leishmaniasis (CVL) serodiagnosis [11, 12]. Among these antigens, the recombinant protein rLci5 demonstrated promising results when used in an enzyme-linked immunosorbent assay (ELISA), offering 87% sensitivity and 94% specificity, as well as 90% diagnostic accuracy [13]. However, in addition to accuracy, it is also important to assess the ability of diagnostic assays to detect antibodies at early times of infection, as well as to evaluate the persistence of these antibodies in infected dogs, which allows clinicians to perform diagnosis in different phases of infection or stages of disease.

Thus, the present study aimed to evaluate the diagnostic capability of rLci5 ELISA at early stages of natural infection by *L. infantum* in dogs, as well as investigate this protein’s ability to detect persistent infection over a 24-month follow-up period, and then compare the obtained results to those produced by other available diagnostic tests. Canine samples from a cohort study in which natural infection was monitored for 2 years in an endemic area [14] were used for evaluation purposes.

## Methods

### Serum samples and CVL diagnosis

Serum samples were obtained from 48 dogs involved in a prospective 2-year cohort study conducted in Camaçari, Bahia, an area where VL and CVL are endemic [14]. This cohort was followed between February 2014 and November 2017. Dogs were visited at baseline and then revisited at 6-month intervals for testing and evaluation of clinical signs associated with CVL, resulting in a total of five assessments during follow-up. During the cohort study, dogs were tested by an ELISA (EIE-LVC, Bio-Manguinhos, Fiocruz, Brazil), an immunochromatographic assay (DPP-LVC, Bio-Manguinhos, Fiocruz, Brazil), qPCR and cultures of splenic aspirate samples were performed. The following clinical parameters related to CVL were assessed at each time point: nutritional status; mucosa color; periocular dermatitis; ear crusting; ear ulceration; muzzle depigmentation; muzzle hyperkeratosis; muzzle lesions; spleen size; onychogryphosis; alopecia; seborrheic dermatitis; lymphadenomegaly [15]. Scores were assigned in accordance with the presence and intensity of each parameter. A total clinical score was then calculated for each dog at each time point by summing the scores of all parameters. This composite score ranged from 0 to 24 points [15]. All 48 dogs studied herein presented positivity to at least one of the four diagnostic methods previously employed at all evaluated time points during the cohort study.

### rLci5 ELISA and other diagnostic tests

All serum samples previously obtained from the original cohort study were subsequently assessed using rLci5 ELISA as previously described by Borja et al. [13]. Other diagnostic tests were originally employed during the cohort study, including EIE-LVC and DPP-LVC, which were performed in accordance with manufacturer’s specifications. qPCR and cultures of splenic aspirate samples were assessed as described by Rampazzo et al. [16] and Barrouin-Melo et al. [17], respectively. All diagnostic assays were carried out under blinded conditions, that is, investigators interpreted the diagnostic test results obtained from each technique without knowing the other test results for each sample under consideration.

### Evaluation of rLci5 ELISA test performance

#### Comparisons among diagnostic tests and association with clinical score

The results from parasitological, molecular and serological diagnostic testing performed at each time point, for all 48 dogs, were entered in a database created in Excel (Additional file 1: Table S1) and compared statistically (see below).

Clinical scores were assessed for all 48 dogs during the cohort study at each of the five time points. These observations were stratified into three categories: clinical scores in the ranges of 0–3, 4–7, and > 7 (Additional file 1: Table S1). Each of these clinical score categories were evaluated with regard to positivity on each of the five different diagnostic methods.

#### Early positivity

Positivity was initially assigned in each dog by detection of positive results on one or more of four previously employed diagnostic methods (DPP-LVC, EIE-LVC, culture or qPCR). At five time points throughout the follow-up period, dogs were evaluated at no longer than 6-month intervals. The frequencies of the first positive detection were calculated for each diagnostic method at each follow-up period. To assess whether rLci5 ELISA was capable of detecting positivity prior to the other four diagnostic methods, each dog's previous diagnostic test results were compared to those obtained from the in-house antigen ELISA. Early detection was considered when rLci5 ELISA demonstrated positivity at least 6 months before positivity was detected using another method. Positivity frequencies were calculated for a given animal using positive and negative results obtained using each test method.

#### Persistence of positivity on diagnostic tests

The persistence of diagnostic test positivity was evaluated when a given diagnostic test returned positive results for at least two consecutive time points following the initial diagnosis (i.e., over a consecutive 18-month follow-up period consisting of three evaluation time points). Considering the entire 24-month cohort study follow-up period, 18 months corresponded to 75% of the evaluation period.

### Statistical analyses

Frequencies of diagnostic results were calculated using the STATA 12.0 (StataCorp LP, Texas, USA) software program. The unpaired *t*-test was used to compare the average diagnostic test positivity versus the average rLci5 ELISA positivity. The chi-square test was used to compare numbers of positive dogs detected by rLci5 ELISA with those detected by the other diagnostic tests evaluated during the follow-up period.

Positivity on rLci5 ELISA was compared to the other four diagnostic methods using McNemar’s test in accordance with the three different clinical score categories. The persistence of each diagnostic test was calculated, expressed as frequency and then compared to the other methods using McNemar’s test.

All statistical analyses were performed using GraphPad software, and results were considered significant when *P* < 0.05.

## Results

### Frequency of diagnostic test positivity

All 48 dogs included were classified as positive by one of the four diagnostic methods originally employed at the final time point of the cohort study. Of these, 100% tested positive on rLci5 ELISA at least one time point, while 83% (40/48) were positive by qPCR, 75% (36/48) on DPP-LVC, 65% (31/48) on EIE-LVC and 31% (15/48) by culture.

Variable positivity was observed in accordance with each diagnostic test evaluated (Table 1). Parasite cultures presented the lowest rate of positivity, resulting in an average 10% detection rate over the five time points evaluated in the cohort study. qPCR detected an average of 30% of dogs as positive, while DPP-LVC and EIE-LVC averaged 34% and 38%, respectively. rLci5 ELISA detected significantly higher numbers of positive dogs throughout the study, ranging from 46 to 81%, with an average 64% positive detection rate over five evaluation time points (Table 1).

**Table 1:** Frequency of positivity on different diagnostic tests during the 24-month cohort study

Test	Positive result frequency					Average positivity over 24 months (%)	Average positivity versus average rLci5 ELISA positivity (*P* value)**
	Baseline	6 months	12 months	18 months	24 months		
Culture	2/48 (4%)	3/48 (6%)	2/48 (4%)	9/48 (19%)	7/46^a^ (15%)	10	< 0.0001
qPCR	17/48 (35%)	9/48 (19%)	17/48 (35%)	20/48 (42%)	9/48 (19%)	30	0.0034
DPP-LVC	13/48 (27%)	16/48 (33%)	14/48 (29%)	23/48 (48%)	15/48 (31%)	34	0.0045
EIE-LVC	11/48 (23%)	19/48 (40%)	19/48 (40%)	20/48 (42%)	22/48 (46%)	38	0.0105
rLci5 ELISA	25/48 (52%)	39/48 (81%)	31/48 (65%)	22/48 (46%)	37/48 (77%)	64	–

^a^Two samples were discarded due to contamination at 24 months

^**^Unpaired *t*-test

### Early positivity using rLci5 ELISA compared to other diagnostic tests

**Fig. 1:**
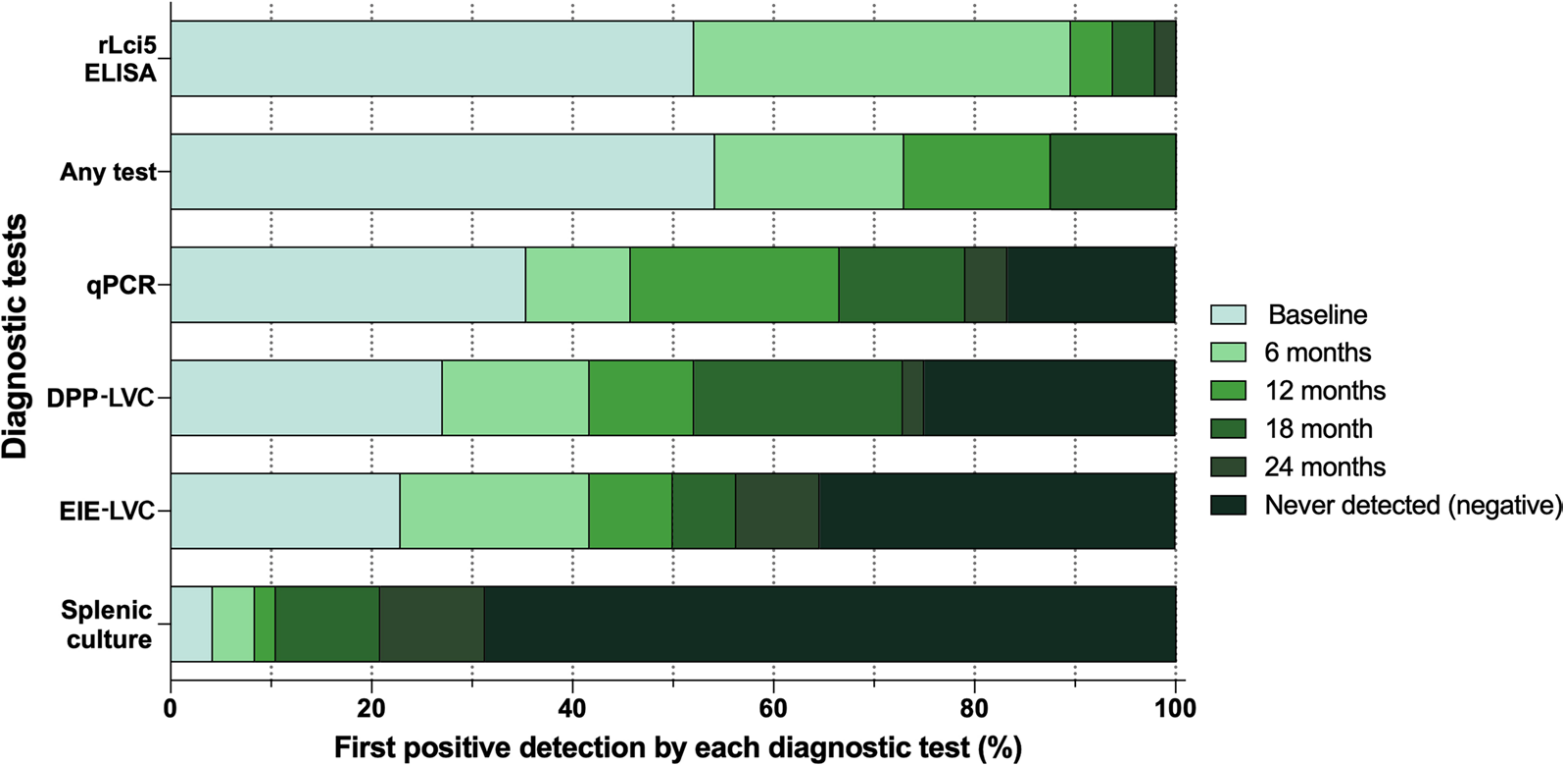
Early positivity to rLci5 ELISA compared to other diagnostic tests during the 24-month cohort study. CVL was initially diagnosed in each dog by positivity on one or more of four previously employed diagnostic methods (DPP-LVC, EIE-LVC, culture or qPCR) and then by rLci5 ELISA. Forty-eight dogs were evaluated at five time points (represented by different shades of green) throughout the follow-up period with 6-month intervals. The frequencies of the first positive detection were calculated for each diagnostic method at each follow-up period

rLci5 ELISA presented higher rates of positivity at early time points compared to the other diagnostic tests employed in the cohort study, as early as 24 months prior to detection by other tests. Our analysis of positive detection revealed first positivity by rLci5 ELISA in most dogs at earlier time points compared to other diagnostic methods (Fig. 1). rLci5 ELISA was positive in 52.1% of the dogs (25/48) at baseline, while qPCR detected 35.4% (17/48), DPP_LVC 27.1% (13/48), EIE-LVC 22.9% (11/48) and culture showed just 4.2% (2/48) positivity (Fig. 1).

**Fig. 2:**
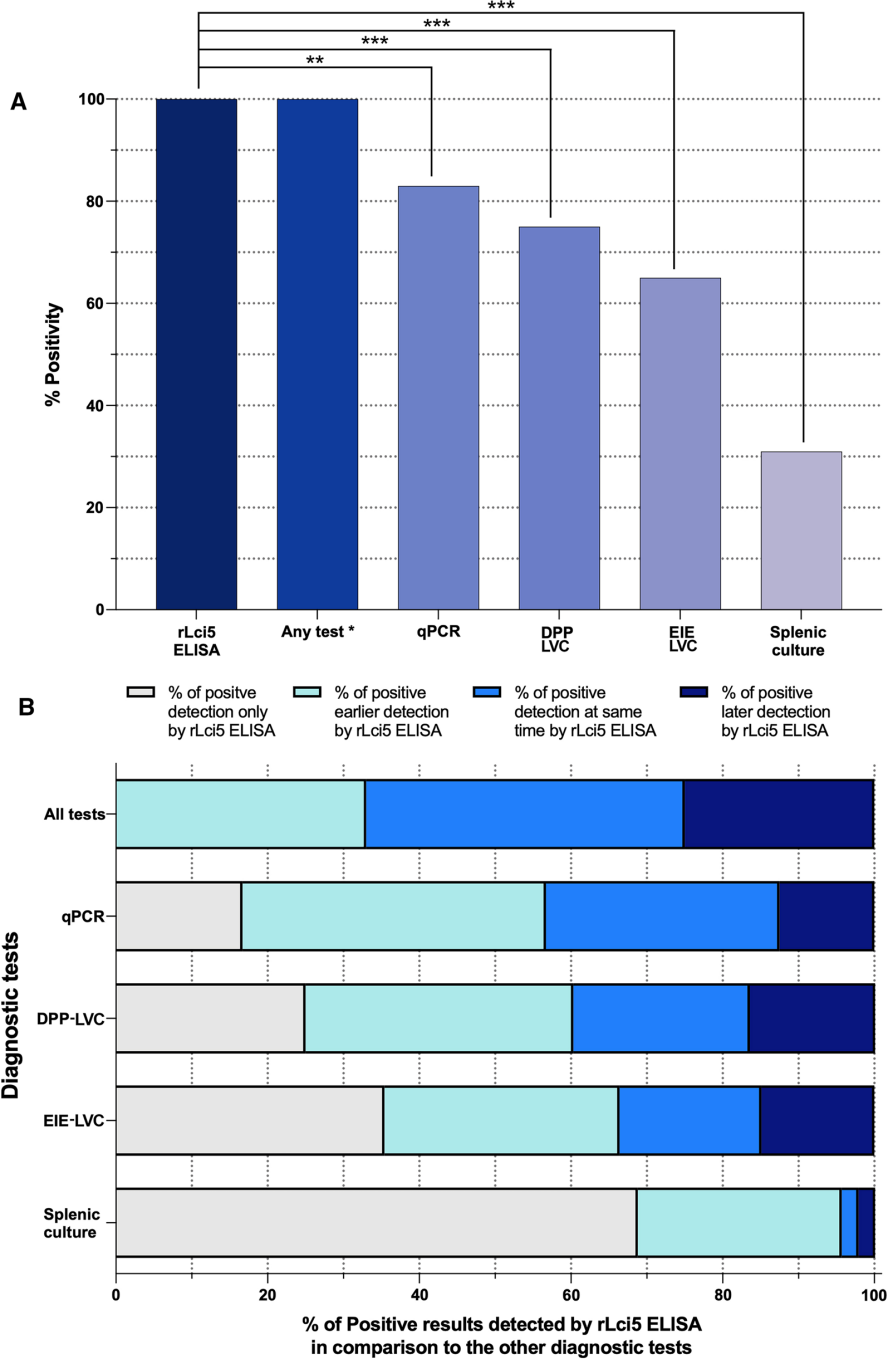
Frequency of diagnostic test positivity by rLci5 ELISA in comparison to other diagnostic methods. **a** Overall positivity rates of each diagnostic test evaluated. The chi-square test was used to compare numbers of positive dogs detected by rLci5 ELISA with those detected by the other diagnostic tests evaluated during the follow-up period. Results were considered significant when *P* < *0.05*. **b** Percentage of early (at least 6 months earlier), simultaneous and later (6 months later or more) detection by rLci5 compared to other diagnostic methods evaluated

With respect to positivity in each of the 48 dogs with CVL, rLci5 ELISA demonstrated positivity in 100% (48/48) of the dogs in at least one of the time points of the cohort study, while qPCR detected 83% (40/48), DPP-LVC 75% (36/48) and EIE LVC 65% (31/48), and culture presented an overall positivity in 31% (15/48) of the animals (Fig. 2a). Moreover, rLci5 ELISA detected positive results earlier than the other diagnostic tests, in 86% (13/15) of the dogs detected by culture, and in almost 50% of the dogs detected by EIE-LVC, DPP-LVC or qPCR (Fig. 2b).

### Associations between clinical scores and diagnostic methods results

With respect to all evaluations (*n* = 240) conducted in the 48 animals during the 24-month cohort study, 62.9% (151/240) were classified as clinical score 0–3, 29.1% (70/240) as clinical score 4–7, and 7.9% (19/240) as clinical score > 7.

**Fig. 3:**
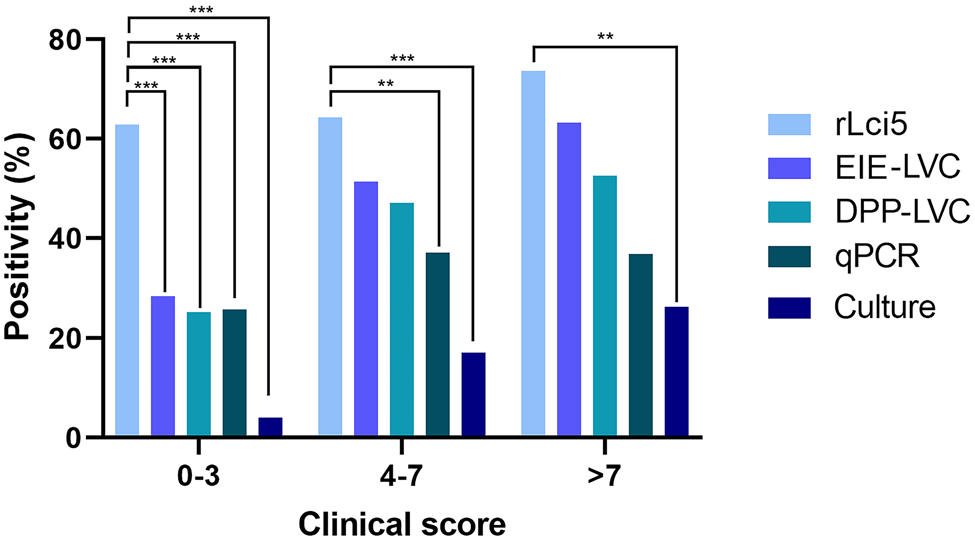
Frequency of diagnostic test positivity in association with overall clinical score. Clinical scores were assessed for all 48 dogs during the cohort study at each of the five time points. These observations were stratified into three categories: clinical scores in the range of 0–3, 4–7, and > 7. Each of these clinical score categories were evaluated with regard to positivity on each of the five different diagnostic methods (different shades of blue). Positivity on rLci5 ELISA was compared to the other four diagnostic methods using McNemar’s test in accordance with the three different clinical score categories (**P < 0.01; ***P < 0.001)

rLci5 ELISA successfully detected positivity in 62.9% (95/151) of the evaluations classified as clinical score 0–3, 64.3% (45/70) as clinical score 4–7, and 73.7% (14/19) as clinical score > 7. Correspondingly, EIE-LVC detected 28.5% (43/151) of the evaluations classified as clinical score 0–3, 51.4% (36/70) as clinical score 4–7, and 63.2% (12/19) as clinical score > 7. DPP-LVC detected 25.2% (38/151) of the evaluations classified as clinical score 0–3, 47.1% (33/70) as clinical score 4–7, and 52.6% (10/19) as clinical score > 7 (Fig. 3). qPCR detected in 25.8% (39/151) of the evaluations classified as clinical score 0–3; 37.1% (26/70) as clinical score 4–7, and 36.8% (7/19) as clinical score > 7. Lastly, parasitological culture detected 4% (6/149) of the evaluations classified as clinical score 0–3, 17.1% (12/70) as clinical score 4–7, and 26.3% (5/19) as clinical score > 7 (Fig. 3). Overall, rLci5 ELISA presented significantly higher detection rates in animals classified with a clinical score of 0–3 compared to the other diagnostic tests evaluated (***P < 0.001). For the dogs with clinical scores ranging from 4 to 7, rLci5 ELISA presented significantly higher detection rates than culture (***P < 0.001) or qPCR (**P < 0.01). Finally, in dogs with clinical scores > 7, rLci5 ELISA only demonstrated significantly higher rates of detection than culture (**P < 0.01).

### Persistence of positivity on different diagnostic tests

**Fig. 4:**
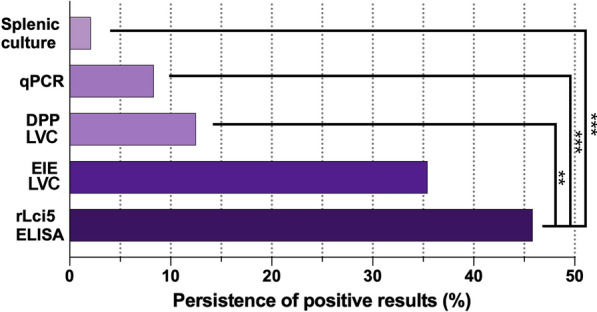
Persistence of positivity to rLci5 ELISA compared to other diagnostic tests during the 24-month cohort study. The persistence of diagnostic test positivity was evaluated when a given diagnostic test returned positive results for at least two consecutive time points following the initial diagnosis. The persistence of each diagnostic test was calculated, expressed as frequency and then compared to the other methods using McNemar’s test (**P < 0.01; ***P < 0.001)

rLci5 ELISA was found to consistently present significantly greater persistent positivity compared to the other diagnostic tests evaluated (*P* < 0.01), with the exception of EIE-LVC. The highest rate of persistence with respect to positivity was identified for rLci5 ELISA (45.8% of the dogs followed up), while EIE-LVC presented a 35.4% rate of persistence compared to DPP-LVC, with 12.5% persistence. Parasitological and molecular methods demonstrated the lowest rates of persistence: 8.3% for qPCR and 2.1% for culture (Fig. 4).

## Discussion

Many factors can influence the accuracy of disease diagnosis. The evolution of a given disease may vary, and it is expected that after infection, biochemical and physiological reactions take place, yet often without evident symptoms. Each disease presents a different course of evolution over time, with variability in signs, symptoms and outcomes [18].

Veterinarians experience difficulty in the assessment of CVL diagnosis due to the non-specificity of clinical signs, which can be confounded with other diseases, such as ehrlichiosis and babesiosis [10]. Moreover, animals considered resistant to CVL may never present any signs characteristic of disease, even over prolonged periods of monitoring [19].

The obtainment of early, rapid and efficient diagnoses is essential to proper clinical treatment and effective control of *Leishmania* transmission, as control is highly dependent on the accurate diagnosis of infected reservoirs [20]. While qPCR could be considered the most efficient method of CVL diagnosis, since it presents elevated sensitivity and specificity, this method can be expensive, and accuracy is highly dependent on sample source [18, 21, 22]. Different samples from bone marrow and lymph node have been used extensively, but spleen aspirate collection, although an invasive method, provides the most accurate detection of infection using qPCR [23]. Culture, considered the gold standard for CVL diagnosis, lacks sensitivity, and is very time-consuming [23]. Alternatively, serological methods are less expensive and more time-efficient. Although the Brazilian Ministry of Health recommends both DPP-LVC and EIE-LVC to assess *L. infantum* infection in dogs, such tests have demonstrated poor results compared to other methods. In a study performed by Laurenti et al. [24], DPP-LVC showed a sensitivity of 90.6% and specificity of 95.1% and presented 44% cross-reactivity with the sera of dogs with babesiosis. EIE-LVC showed a good sensitivity (90.6%), but very low specificity (77.8%) due to the high cross-reactivity with the sera from the animals harboring other pathogens [24]. More recently, Teixeira et al. [25] evaluated both DPP-LVC and EIE-LVC based on the use of asymptomatic reference standard samples previously confirmed by parasitological and molecular techniques. Their study detected sensitivity and specificity for DPP-LVC and EIE-LVC of 21.7% and 92.6%, and 11.6% and 90.7%, respectively [25]. Thus, in an effort to improve the accuracy of CVL diagnosis in Brazil, the implementation of more reliable test methods is recommended [26, 27].

Recombinant protein rLci5 previously demonstrated promise in CVL serodiagnosis, showing high sensitivity and specificity [13]. The present study confirmed the accuracy of rLci5 ELISA in CVL serodiagnosis, as this test presented the highest detection rate among the diagnostic tests evaluated. Moreover, rLci5 ELISA was observed to detect positive results earlier when compared to the methods currently recommended by the Brazilian Ministry of Health (DPP-LVC, EIE-LVC), as well as qPCR and culture. The early detection capability of rLci5 ELISA represents a promising finding, as the adoption of diagnostic methods capable of detecting the presence of anti-*Leishmania* antibodies shortly after the onset of infection are well-suited to control transmission [28]. Accordingly, rLci5 ELISA warrants consideration by health authorities to improve the control of CVL transmission, and could make a valuable contribution together with other control measures to preclude the spread of this disease.

qPCR demonstrated the highest positive rate among the tests evaluated (except for rLci5). However, its performance was highly variable among the time points evaluated (Fig. 4). qPCR is a very sensitive technique, but its results strongly depend on the samples and tissues used. Even using splenic tissue samples, which was shown to be one of the best tissues to measure parasite load in dogs [16, 23], it has already been demonstrated that the region of the spleen where the samples were obtained can influence the positivity of qPCR [29]. Recently our group showed that in the same dog followed in a cohort study, there was significant variation in positivity and parasite load measured by qPCR at different points evaluated [14].

As expected, the positivity rates of all five diagnostic tests evaluated in this study increased in accordance with the severity of clinical manifestations. However, rLci5 ELISA offered favorable detection rates among even those dogs with lower clinical scores (Fig. 3), as demonstrated previously [13], which reinforces the superiority of rLci5 ELISA alone in comparison to other test methods. The ability of rLci5 ELISA to test positive earlier than other methods could be explained by reactivity to the humoral response in dogs, even when clinical signs are not readily apparent. It is therefore possible that while parasite load may not be high enough to be detected by qPCR or culture, it could be sufficient to stimulate a humoral response that is detectable by rLci5 ELISA.

The obtainment of persistent results throughout the course of infection confers robustness to diagnosis and reduces the risk of false-positive and false-negative results depending on the time of analysis. rLci5 ELISA showed a significantly higher persistence of positivity during the follow-up period compared to other diagnostic tests evaluated. While the literature contains longitudinal studies evaluating CVL diagnostic methods, none have attempted to assess the persistence of positive results in a longitudinal manner [30–32]. Importantly, other studies have evaluated the persistence of diagnostic test results in diseases other than leishmaniasis, such as allergic contact dermatitis, asthma and rheumatoid arthritis [33–35].

Diagnostic test results can vary over time, likely due to a range of physiological processes associated with disease that affect the host [36]. Titers of antibodies can be low or high at different stages of disease [36], and parasites may not be detectable in culture due to low parasite burden [37]; moreover, the detection of parasite DNA can vary among tissue types under PCR testing [23, 29]. Our findings demonstrate considerable variation in the diagnostic results produced by culture and qPCR over time compared to serological testing. Studies have shown that the persistence of antibodies for a considerable period of time may favor CVL diagnosis using serological methods [31]. On the other hand, parasite load is highly dependent on the type of sample used, the presence of PCR inhibitors and natural variations throughout the course of infection that are dependent on an animal's individual immune response. Further studies may serve to clarify the relationship between the persistence of diagnostic test results and specific pathological processes occurring within the host.

## Conclusion

rLci5 ELISA demonstrated a capability to achieve earlier positivity, with higher persistence of positive results than DPP-LVC, qPCR or culture. The persistence of positivity in the same animals throughout the study period revealed this diagnostic test’s consistency and reliability. In sum, rLci5 ELISA was shown to be an important tool for CVL diagnosis, providing stable results over time and fewer false-negative results along the course of natural infection. We recommend that health authorities consider the use of rLci5 in ELISA to improve the performance of CVL diagnosis.

## Supplementary Information

**Additional file 1: Table S1**. Diagnostic results and clinical classification for 48 dogs at each time point of the study.

## Data Availability

All data generated or analyzed during this study are included in this published article and its supplementary information file (Additional file 1: Table S1).

## Abbreviations

DNA: Deoxyribonucleic acid
DPP: Dual Path Platform
EIE: Immunoenzymatic assay
ELISA: Enzyme linked immunosorbent assay
IGM: Gonçalo Moniz Institute
VL: Visceral leishmaniasis
CVL: Canine visceral leishmaniasis
PCR: Polymerase chain reaction
qPCR: Real-time or quantitative PCR
WHO: World Health Organization
