# Supplementary material for: Early detection and persistent positivity of anti-Leishmania antibodies using a recombinant protein-based ELISA in naturally infected dogs in Brazil
Source: Parasit Vectors. 2021 Aug 12;14:398. doi: 10.1186/s13071-021-04895-z (PMC8359089; doi:10.1186/s13071-021-04895-z)
Supplement: Supplementary file 1 — Additional file 1: Table S1. Diagnostic results and clinical classification for 48 dogs at each time point of the study. [file 13071_2021_4895_MOESM1_ESM.pdf]

Additional file 1: Table S1. Diagnostic results and clinical classification for 48 dogs at each timepoint of the study.

Sample ID	Months	DPP	EIE	qPCR	Culture	rLCI5	Res_All_tests (rlci5 not included)	Clinical Classification
RUR 186	24	POS	POS	NEG	NEG	POS	POS	>7
RUR 273	18	POS	POS	POS	POS	POS	POS	>7
RUR 067	0	POS	NEG	POS	NEG	POS	POS	4 to 7
RUR 186	18	POS	POS	NEG	NEG	POS	POS	>7
RUR 211	12	NEG	NEG	NEG	NEG	POS	NEG	>7
RUR 070	0	NEG	NEG	POS	NEG	POS	POS	4 to 7
RUR 273	24	POS	POS	POS	POS	POS	POS	>7
RUR 186	12	NEG	POS	NEG	NEG	POS	NEG	>7
RUR 218	18	POS	NEG	POS	NEG	NEG	POS	>7
RUR 186	6	POS	POS	NEG	NEG	POS	POS	>7
RUR 099	6	NEG	POS	NEG	NEG	POS	NEG	>7
RUR 211	24	POS	POS	POS	POS	POS	POS	>7
RUR 218	24	POS	POS	POS	POS	POS	POS	>7
RUR 241	18	POS	NEG	NEG	NEG	POS	NEG	>7
RUR 241	24	POS	POS	NEG	NEG	NEG	POS	>7
RUR 083	0	NEG	NEG	NEG	NEG	POS	NEG	0 to 3
RUR 368	24	NEG	POS	NEG	POS	POS	POS	>7
RUR 453	12	NEG	NEG	POS	NEG	NEG	POS	>7
RUR 471	18	NEG	POS	NEG	NEG	POS	NEG	>7
RUR 099	24	NEG	POS	NEG	NEG	POS	NEG	4 to 7
RUR 106	24	NEG	POS	NEG	NEG	POS	NEG	4 to 7
RUR 218	6	NEG	NEG	NEG	NEG	NEG	NEG	4 to 7
RUR 218	12	NEG	NEG	NEG	NEG	POS	NEG	4 to 7
RUR 403	24	POS	POS	NEG	NEG	POS	POS	4 to 7
RUR 454	12	NEG	NEG	NEG	NEG	NEG	NEG	4 to 7
RUR 471	12	POS	POS	POS	NEG	NEG	POS	4 to 7
RUR 067	6	POS	POS	POS	POS	POS	POS	4 to 7
RUR 070	12	NEG	NEG	POS	NEG	POS	POS	4 to 7

RUR 099	18	NEG	POS	NEG	NEG	NEG	NEG	4 to 7
RUR 185	24	POS	POS	POS	POS	POS	POS	4 to 7
RUR 221	18	NEG	NEG	NEG	NEG	NEG	NEG	4 to 7
RUR 241	6	NEG	NEG	NEG	NEG	POS	NEG	4 to 7
RUR 273	12	NEG	NEG	NEG	NEG	POS	NEG	4 to 7
RUR 274	12	NEG	NEG	NEG	NEG	NEG	NEG	4 to 7
RUR 329	18	POS	NEG	NEG	NEG	POS	NEG	4 to 7
RUR 367	24	POS	POS	NEG	NEG	POS	POS	4 to 7
RUR 084	0	NEG	POS	NEG	NEG	NEG	NEG	0 to 3
RUR 403	12	POS	POS	POS	NEG	POS	POS	4 to 7
RUR 403	18	POS	POS	POS	POS	NEG	POS	4 to 7
RUR 099	0	NEG	POS	POS	NEG	POS	POS	0 to 3
RUR 106	0	POS	POS	NEG	NEG	NEG	POS	0 to 3
RUR 067	18	POS	POS	POS	POS	NEG	POS	4 to 7
RUR 141	12	NEG	POS	POS	NEG	POS	POS	4 to 7
RUR 115	0	POS	NEG	NEG	NEG	NEG	NEG	4 to 7
RUR 116	0	POS	NEG	NEG	NEG	POS	NEG	0 to 3
RUR 153	24	POS	POS	NEG	NEG	POS	POS	4 to 7
RUR 184	18	NEG	POS	NEG	NEG	NEG	NEG	4 to 7
RUR 329	6	NEG	POS	NEG	NEG	POS	NEG	4 to 7
RUR 123	0	NEG	NEG	POS	NEG	NEG	POS	0 to 3
RUR 364	18	NEG	POS	NEG	NEG	POS	NEG	4 to 7
RUR 403	6	POS	NEG	POS	POS	POS	POS	4 to 7
RUR 418	6	NEG	POS	POS	NEG	POS	POS	4 to 7
RUR 140	0	NEG	NEG	POS	NEG	NEG	POS	0 to 3
RUR 067	24	POS	POS	NEG	NEG	NEG	POS	4 to 7
RUR 141	0	POS	POS	POS	POS	POS	POS	4 to 7
RUR 070	24	NEG	NEG	NEG	NEG	NEG	NEG	4 to 7
RUR 099	12	NEG	POS	NEG	NEG	POS	NEG	4 to 7
RUR 106	18	POS	POS	NEG	NEG	POS	POS	4 to 7
RUR 145	0	NEG	POS	POS	NEG	NEG	POS	4 to 7

RUR 116	6	POS	NEG	NEG	NEG	POS	NEG	4 to 7
RUR 123	18	POS	POS	NEG	NEG	NEG	POS	4 to 7
RUR 140	18	NEG	NEG	NEG	NEG	NEG	NEG	4 to 7
RUR 140	24	NEG	NEG	NEG	NEG	POS	NEG	4 to 7
RUR 147	0	NEG	NEG	POS	NEG	POS	POS	4 to 7
RUR 141	6	POS	POS	POS	POS	POS	POS	4 to 7
RUR 141	18	NEG	NEG	POS	POS	NEG	POS	4 to 7
RUR 141	24	POS	POS	POS	NEG	POS	POS	4 to 7
RUR 145	12	NEG	NEG	NEG	NEG	POS	NEG	4 to 7
RUR 145	24	NEG	NEG	NEG	NEG	POS	NEG	4 to 7
RUR 185	18	POS	POS	POS	NEG	NEG	POS	4 to 7
RUR 211	6	NEG	POS	NEG	NEG	POS	NEG	4 to 7
RUR 211	18	POS	NEG	NEG	NEG	POS	NEG	4 to 7
RUR 153	0	POS	POS	POS	NEG	POS	POS	0 to 3
RUR 273	6	NEG	NEG	POS	NEG	POS	POS	4 to 7
RUR 274	18	POS	NEG	POS	POS	POS	POS	4 to 7
RUR 329	24	NEG	POS	NEG	NEG	POS	NEG	4 to 7
RUR 361	12	NEG	NEG	POS	NEG	POS	POS	4 to 7
RUR 362	6	POS	POS	NEG	NEG	POS	POS	4 to 7
RUR 362	12	POS	POS	POS	NEG	POS	POS	4 to 7
RUR 362	18	NEG	POS	POS	POS	NEG	POS	4 to 7
RUR 362	24	POS	NEG	NEG	NEG	POS	NEG	4 to 7
RUR 390	12	POS	POS	POS	POS	POS	POS	4 to 7
RUR 399	12	NEG	NEG	NEG	NEG	NEG	NEG	4 to 7
RUR 431	12	POS	POS	NEG	NEG	NEG	POS	4 to 7
RUR 431	18	POS	POS	NEG	NEG	NEG	POS	4 to 7
RUR 433	18	POS	NEG	POS	POS	NEG	POS	4 to 7
RUR 453	6	NEG	NEG	NEG	NEG	POS	NEG	4 to 7
RUR 453	18	NEG	POS	NEG	NEG	POS	NEG	4 to 7
RUR 456	12	NEG	NEG	NEG	NEG	POS	NEG	4 to 7
RUR 067	12	POS	POS	POS	POS	NEG	POS	0 to 3

RUR 070	6	NEG	NEG	POS	NEG	POS	POS	0 to 3
RUR 184	0	NEG	NEG	NEG	NEG	POS	NEG	0 to 3
RUR 084	24	NEG	POS	NEG	NEG	POS	NEG	0 to 3
RUR 185	0	POS	NEG	POS	NEG	POS	POS	0 to 3
RUR 106	6	POS	POS	NEG	NEG	POS	POS	0 to 3
RUR 186	0	NEG	NEG	POS	NEG	POS	POS	>7
RUR 116	12	NEG	NEG	NEG	NEG	POS	NEG	0 to 3
RUR 116	24	NEG	NEG	NEG	NEG	NEG	NEG	0 to 3
RUR 123	24	NEG	POS	NEG	NEG	POS	NEG	0 to 3
RUR 147	6	NEG	NEG	NEG	NEG	NEG	NEG	0 to 3
RUR 147	12	NEG	NEG	NEG	NEG	NEG	NEG	0 to 3
RUR 185	6	POS	POS	NEG	NEG	POS	POS	0 to 3
RUR 211	0	NEG	POS	NEG	NEG	POS	NEG	0 to 3
RUR 221	12	NEG	NEG	POS	NEG	POS	POS	0 to 3
RUR 221	24	NEG	NEG	NEG	NEG	NEG	NEG	0 to 3
RUR 233	6	NEG	POS	NEG	NEG	NEG	NEG	0 to 3
RUR 233	12	NEG	POS	NEG	NEG	NEG	NEG	0 to 3
RUR 241	12	NEG	NEG	POS	NEG	POS	POS	0 to 3
RUR 274	6	NEG	NEG	NEG	NEG	POS	NEG	0 to 3
RUR 364	12	NEG	POS	NEG	NEG	POS	NEG	0 to 3
RUR 367	12	NEG	NEG	POS	NEG	POS	POS	0 to 3
RUR 367	18	NEG	NEG	NEG	NEG	POS	NEG	0 to 3
RUR 218	0	NEG	NEG	NEG	NEG	NEG	NEG	7 plu>7
RUR 368	12	NEG	NEG	NEG	NEG	POS	NEG	0 to 3
RUR 368	18	NEG	NEG	POS	NEG	NEG	POS	0 to 3
RUR 376	24	NEG	NEG	NEG	NEG	POS	NEG	0 to 3
RUR 384	18	NEG	NEG	POS	POS	POS	POS	0 to 3
RUR 390	6	NEG	POS	NEG	NEG	POS	NEG	0 to 3
RUR 390	24	NEG	NEG	NEG	NEG	POS	NEG	0 to 3
RUR 402	18	NEG	NEG	POS	NEG	NEG	POS	0 to 3
RUR 418	12	NEG	NEG	NEG	NEG	NEG	NEG	0 to 3

RUR 221	0	NEG	NEG	NEG	NEG	NEG	NEG	0 to 3
RUR 432	18	NEG	NEG	NEG	NEG	NEG	NEG	0 to 3
RUR 433	12	POS	NEG	NEG	NEG	POS	NEG	0 to 3
RUR 454	6	NEG	NEG	NEG	NEG	NEG	NEG	0 to 3
RUR 466	18	NEG	NEG	NEG	NEG	POS	NEG	0 to 3
RUR 471	6	POS	NEG	NEG	NEG	POS	NEG	0 to 3
RUR 070	18	NEG	NEG	POS	NEG	NEG	POS	0 to 3
RUR 084	12	NEG	POS	NEG	NEG	POS	NEG	0 to 3
RUR 106	12	POS	POS	NEG	NEG	POS	POS	0 to 3
RUR 115	6	POS	POS	NEG	NEG	POS	POS	0 to 3
RUR 115	24	NEG	POS	POS	NEG	NEG	POS	0 to 3
RUR 233	0	NEG	NEG	POS	NEG	NEG	POS	0 to 3
RUR 140	6	NEG	POS	NEG	NEG	POS	NEG	0 to 3
RUR 145	18	NEG	NEG	POS	NEG	POS	POS	0 to 3
RUR 147	18	POS	NEG	NEG	NEG	POS	NEG	0 to 3
RUR 147	24	NEG	NEG	NEG	NEG	NEG	NEG	0 to 3
RUR 241	0	NEG	NEG	NEG	NEG	NEG	NEG	4 to 7
RUR 153	12	NEG	POS	NEG	NEG	NEG	NEG	0 to 3
RUR 184	12	NEG	POS	POS	NEG	POS	POS	0 to 3
RUR 243	0	NEG	NEG	NEG	NEG	NEG	NEG	0 to 3
RUR 233	24	NEG	POS	NEG	NEG	POS	NEG	0 to 3
RUR 274	24	NEG	NEG	POS	POS	POS	POS	0 to 3
RUR 329	12	POS	POS	NEG	NEG	POS	POS	0 to 3
RUR 273	0	NEG	NEG	NEG	NEG	NEG	NEG	>7
RUR 367	6	NEG	NEG	NEG	NEG	POS	NEG	0 to 3
RUR 274	0	NEG	NEG	NEG	NEG	NEG	NEG	0 to 3
RUR 376	6	NEG	NEG	NEG	NEG	POS	NEG	0 to 3
RUR 329	0	POS	POS	NEG	POS	NEG	POS	0 to 3
RUR 384	6	NEG	NEG	NEG	NEG	POS	NEG	0 to 3
RUR 384	12	POS	NEG	POS	NEG	POS	POS	0 to 3
RUR 390	18	NEG	POS	POS	POS	POS	POS	0 to 3

RUR 361	0	NEG	NEG	NEG	NEG	NEG	NEG	0 to 3
RUR 362	0	POS	POS	NEG	NEG	NEG	POS	4 to 7
RUR 402	24	NEG	NEG	NEG	NEG	POS	NEG	0 to 3
RUR 418	18	NEG	NEG	POS	NEG	POS	POS	0 to 3
RUR 418	24	NEG	NEG	NEG	NEG	POS	NEG	0 to 3
RUR 364	0	NEG	NEG	NEG	NEG	NEG	NEG	0 to 3
RUR 420	24	NEG	NEG	NEG	NEG	POS	NEG	0 to 3
RUR 431	6	NEG	POS	NEG	NEG	POS	NEG	0 to 3
RUR 431	24	NEG	POS	NEG	NEG	POS	NEG	0 to 3
RUR 367	0	NEG	NEG	NEG	NEG	POS	NEG	0 to 3
RUR 432	6	NEG	NEG	NEG	NEG	POS	NEG	0 to 3
RUR 432	24	NEG	NEG	NEG	NEG	POS	NEG	0 to 3
RUR 368	0	NEG	NEG	NEG	NEG	POS	NEG	0 to 3
RUR 453	24	NEG	NEG	NEG	NEG	POS	NEG	0 to 3
RUR 454	18	NEG	NEG	NEG	NEG	POS	NEG	0 to 3
RUR 456	18	NEG	POS	NEG	NEG	POS	NEG	0 to 3
RUR 466	12	POS	NEG	NEG	NEG	NEG	NEG	0 to 3
RUR 466	24	NEG	NEG	NEG	NEG	POS	NEG	0 to 3
RUR 471	24	NEG	NEG	NEG	NEG	POS	NEG	0 to 3
RUR 083	18	POS	NEG	NEG	NEG	NEG	NEG	0 to 3
RUR 083	24	POS	NEG	NEG	NEG	NEG	NEG	0 to 3
RUR 084	6	POS	POS	POS	NEG	POS	POS	0 to 3
RUR 084	18	NEG	POS	POS	NEG	NEG	POS	0 to 3
RUR 115	12	NEG	POS	NEG	NEG	POS	NEG	0 to 3
RUR 115	18	POS	POS	NEG	NEG	NEG	POS	0 to 3
RUR 116	18	POS	NEG	NEG	NEG	NEG	NEG	0 to 3
RUR 123	12	NEG	NEG	NEG	NEG	NEG	NEG	0 to 3
RUR 140	12	NEG	NEG	POS	NEG	POS	POS	0 to 3
RUR 145	6	NEG	NEG	NEG	NEG	NEG	NEG	0 to 3
RUR 153	6	POS	POS	NEG	NEG	POS	POS	0 to 3
RUR 184	6	NEG	POS	NEG	NEG	POS	NEG	0 to 3

RUR 184	24	NEG	NEG	NEG	NEG	NEG	NEG	0 to 3
RUR 376	0	NEG	NEG	POS	NEG	POS	POS	0 to 3
RUR 185	12	NEG	POS	NEG	NEG	POS	NEG	0 to 3
RUR 384	0	NEG	NEG	NEG		POS	NEG	0 to 3
RUR 233	18	POS	POS	NEG	NEG	NEG	POS	0 to 3
RUR 390	0	POS	POS	POS	NEG	NEG	POS	0 to 3
RUR 243	12	POS	POS	POS	NEG	POS	POS	0 to 3
RUR 399	0	NEG	NEG	NEG	NEG	POS	NEG	0 to 3
RUR 361	18	NEG	NEG	NEG	NEG	POS	NEG	0 to 3
RUR 361	24	NEG	NEG	NEG	NEG	POS	NEG	0 to 3
RUR 402	0	NEG	NEG	NEG	NEG	POS	NEG	0 to 3
RUR 364	24	NEG	POS	NEG	NEG	POS	NEG	0 to 3
RUR 368	6	POS	NEG	NEG	NEG	NEG	NEG	0 to 3
RUR 376	12	POS	NEG	NEG	NEG	NEG	NEG	0 to 3
RUR 376	18	POS	NEG	POS	NEG	POS	POS	0 to 3
RUR 384	24	NEG	NEG	NEG		POS	NEG	0 to 3
RUR 399	6	NEG	NEG	NEG	NEG	POS	NEG	0 to 3
RUR 399	18	POS	NEG	POS	NEG	NEG	POS	0 to 3
RUR 399	24	NEG	NEG	NEG	NEG	NEG	NEG	0 to 3
RUR 402	12	NEG	NEG	NEG	NEG	NEG	NEG	0 to 3
RUR 432	12	POS	NEG	NEG	NEG	POS	NEG	0 to 3
RUR 403	0	POS	NEG	NEG	NEG	POS	NEG	4 to 7
RUR 433	6	POS	NEG	NEG	NEG	POS	NEG	0 to 3
RUR 433	24	POS	NEG	POS	NEG	POS	POS	0 to 3
RUR 454	24	NEG	NEG	NEG	NEG	POS	NEG	0 to 3
RUR 418	0	NEG	NEG	NEG	NEG	NEG	NEG	0 to 3
RUR 466	6	NEG	NEG	NEG	NEG	NEG	NEG	0 to 3
RUR 420	0	NEG	NEG	NEG	NEG	POS	NEG	0 to 3
RUR 469	6	*NEG*	NEG	POS	NEG	POS	POS	0 to 3
RUR 469	18	NEG	NEG	NEG	NEG	POS	NEG	0 to 3
RUR 469	24	NEG	NEG	NEG	NEG	POS	NEG	0 to 3

RUR 431	0	POS	POS	POS	NEG	POS	POS	0 to 3
RUR 083	6	NEG	NEG	NEG	NEG	NEG	NEG	0 to 3
RUR 083	12	NEG	NEG	NEG	NEG	NEG	NEG	0 to 3
RUR 432	0	NEG	NEG	NEG	NEG	POS	NEG	0 to 3
RUR 123	6	NEG	POS	NEG	NEG	POS	NEG	0 to 3
RUR 433	0	NEG	NEG	NEG	NEG	NEG	NEG	0 to 3
RUR 153	18	POS	POS	POS	NEG	NEG	POS	0 to 3
RUR 453	0	NEG	NEG	NEG	NEG	NEG	NEG	0 to 3
RUR 221	6	NEG	NEG	POS	NEG	POS	POS	0 to 3
RUR 243	6	NEG	NEG	NEG	NEG	POS	NEG	0 to 3
RUR 243	18	POS	NEG	NEG	NEG	NEG	NEG	0 to 3
RUR 243	24	POS	POS	POS	POS	NEG	POS	0 to 3
RUR 454	0	POS	NEG	NEG	NEG	NEG	NEG	4 to 7
RUR 456	0	NEG	NEG	NEG	NEG	POS	NEG	0 to 3
RUR 361	6	POS	NEG	NEG	NEG	POS	NEG	0 to 3
RUR 364	6	POS	NEG	NEG	NEG	POS	NEG	0 to 3
RUR 466	0	NEG	NEG	POS	NEG	NEG	POS	0 to 3
RUR 402	6	NEG	NEG	NEG	NEG	POS	NEG	0 to 3
RUR 469	0	NEG	NEG	POS	NEG	POS	POS	0 to 3
RUR 420	6	NEG	NEG	NEG	NEG	POS	NEG	0 to 3
RUR 420	12	NEG	NEG	POS	NEG	NEG	POS	0 to 3
RUR 420	18	NEG	NEG	NEG	NEG	NEG	NEG	0 to 3
RUR 471	0	NEG	NEG	NEG	NEG	POS	NEG	4 to 7
RUR 456	6	NEG	NEG	NEG	NEG	NEG	NEG	0 to 3
RUR 456	24	NEG	NEG	NEG	NEG	POS	NEG	0 to 3
RUR 469	12	NEG	NEG	NEG	NEG	POS	NEG	0 to 3
